# Use of Tetravalent Galabiose for Inhibition of *Streptococcus Suis* Serotype 2 Infection in a Mouse Model

**DOI:** 10.3390/biology2020702

**Published:** 2013-04-08

**Authors:** Roland J. Pieters, Hans-Christian Slotved, Hanne Møller Mortensen, Lene Arler, Jukka Finne, Sauli Haataja, John A. F. Joosten, Hilbert M. Branderhorst, Karen A. Krogfelt

**Affiliations:** 1 Department of Medicinal Chemistry and Chemical Biology, Utrecht Institute for Pharmaceutical Sciences, Utrecht University, P.O. Box 80082, 3508 TB, Utrecht, The Netherlands; E-Mails: jjoosten@syntarga.com (J.A.F.J.); H.Branderhorst@c-mlabs.com (H.M.B.); 2 Department of Microbiology and Infection Control (MI), Statens Serum Institut, Copenhagen, Denmark; E-Mails: hcs@ssi.dk (H.-C.S.); northern_breeze@hotmail.com (H.M.M.); lenearler@hotmail.com (L.A.); kak@ssi.dk (K.A.K.); 3 Department of Biosciences, Division of Biochemistry and Biotechnology, University of Helsinki, P.O.B. 56, Helsinki FI-00014, Finland; E-Mail: jukka.finne@helsinki.fi; 4 Department of Medical Biochemistry and Genetics, University of Turku, Kiinamyllynkatu 10, Turku FI-20520, Finland; E-Mail: sauhaa@utu.fi

**Keywords:** tetravalent galabiose, *Streptococcus suis*, adhesion inhibition, mouse model

## Abstract

*Streptococcus suis* is an important swine pathogen associated with a variety of infections such as meningitis, arthritis and septicemia. The bacterium is zoonotic and has been found to cause meningitis especially in humans occupationally exposed to infected pigs. Since adhesion is a prerequisite for colonization and subsequent infection, anti-adhesion treatment seems a natural alternative to traditional treatment with antibiotics. In order to optimize the inhibitory potency a multivalency approach was taken in the inhibitor design. A synthetic tetravalent galabiose compound was chosen which had previously shown promising anti-adhesion effects with *S. suis in vitro*. The aim of this study was to evaluate the *in vivo* effects of the compound using an infection peritonitis mouse model. As such *S. suis* serotype 2 infection and treatment were tested *in vivo* and the effects were compared to the effect of treatment with penicillin.

## 1. Introduction

*Streptococcus suis* is a major swine pathogen worldwide and is associated with meningitis, arthritis, endocarditis, septicemia, pneumonia and sudden death especially in young pigs [1]. *S. suis* is considered a zoonotic agent associated with human infections and has especially been described as the cause of meningitis in persons with occupational exposure to pigs [2,3]. *S. suis* is not an obligate pathogen and is commonly isolated from the respiratory tract of pigs [4,5]. So far, at least 33 different serotypes of the bacterium have been described with serotype 2 as the most prevalent serotype isolated from diseased pigs [1,4,6].

Measures taken to control *S. suis* infection have involved treatment with antibiotics, but it is only partly efficient and problems are arising with an increasing development of resistance. The development of an effective vaccine against *S. suis* has not yet been successful due to the lack of knowledge of virulence factors and variability of virulence among serotypes [5,7]. As an alternative to antibiotic treatment and vaccination, the use of a sugar compounds to inhibit bacterial adhesion has been proposed and has actively been pursued for several pathogens [8,9]. Attachment of *S. suis* to host cells is mediated by a recently discovered adhesin SadP [10] that recognizes the galabiose disaccharide galactosyl-(α1-4)-galactose (Galα1-4Gal) on the terminal and internal positions of cell surface glycolipids [11,12]. This galabiose epitope is present in the globoseries of glycolipids on uroepithelial cells and erythrocytes. There are two subtypes of the adhesin P_N_ and P_O_, based on differences in their binding specificity [12,13]. A certain group of pig and human erythrocytes presents a glycolipid containing the Galα(1-4)Galβ(1-4)-Glcβ1-ceramide structure on the surface. These are recognized by the *S. suis* adhesins and in hemagglutination inhibition assays, *S. suis* bacterial cells are mixed with erythrocytes to induce agglutination [14]. In order to develop anti-adhesion based therapeutics, the native carbohydrate ligands need to be modified in order to enhance their potency. This approach has been applied most notably for uropathogenic *Escherichia coli* [15,16] but also for *S. suis* [12]. An alternative approach to improve the inhibitory potencies is multivalency. By linking several copies of the sugar ligand to a core scaffold molecule such as a dendrimer, potencies can be enhanced dramatically [17]. While the strongest effects occur when bridging nearby binding sites is possible by the multivalent ligand (chelate effect), also statistical rebinding effects are significant when such bridging is not possible [17]. Indeed modest benefits for uropathogenic *E. coli* have been observed, e.g. in the recently reported study with cyclodextrin-mannose conjugates [18] and also for galabiose-dendrimer conjugates [19]. Strong multivalency enhancements have been observed in the inhibition of or binding to *S. suis*. Several assay types have confirmed these effects that resulted in IC_50_s in the low nanomolar range, for systems with valencies ranging from two to eight [20,21,22]. The interactions between galabiose and the *S. suis* adhesin were also used in a bacterial detection assay involving magnetic glyconanoparticles with a multivalent display of the sugar ligand [23].

**Figure 1:**
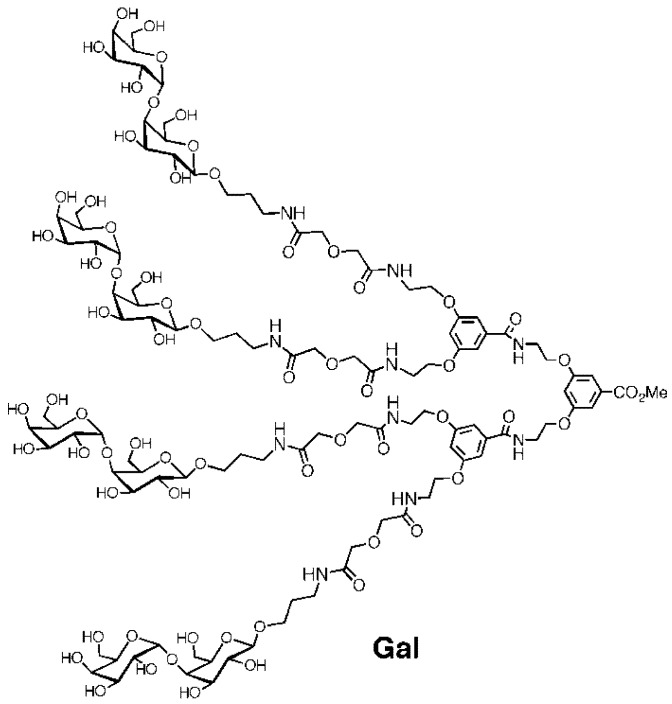
Structure of the tetravalent galabiose compound ‘Gal’.

In order to further explore the potential of the anti-adhesion approach we here present our efforts of using a tetravalent galabiose ligand ('Gal' Figure 1) in an *in vivo* model. A recent *in vivo* study involving monovalent carbohydrate ligands against uropathogenic *E. coli* has shown the potential for the anti-adhesion approach for treatment of urinary tract infections. Furthermore, multivalent carbohydrate ligands were shown to be effective against a bacterial toxin *in vivo* [24]. However, the combination of using multivalent carbohydrates against bacterial adhesion has not yet been reported. The tetravalent 'Gal' was chosen, since it was the most effective compound on a per sugar ligand basis [20,22].

A number of experimental animal models for evaluation of virulence of *S. suis* serotype 2 isolates have been developed in mice and pigs [25]. At present there is no standardized animal model for studying *S. suis* infection, and this has caused some confusion in designating strains as virulent or non-virulent. In several studies, the mouse has proven to be a suitable animal model for the infection with *S. suis* serotype 2, and one of the early experiments by Williams and co-workers determined that the behavior of the bacterium in mice resembled what had previously been reported in pigs [25,26]. Different murine models for the study of *S. suis* infections have been developed involving different experimental setups [27,28,29]. Robertson and Blackmore concluded that the mouse served as the best model to assess pathogenicity of *S. suis* isolates in pigs, since disease was only provoked in mice with isolates pathogenic for pigs [29]. Based on the above we decided to use a mouse model for the examination of *S. suis* serotype 2 infection *in vivo* and, by means of this model, investigate whether the synthetic anti-adhesion compound, the tetravalent ‘Gal’ has an inhibiting effect on the established infection. Furthermore, the model was used to determine the effect of antibiotic treatment of *S. suis* serotype 2 strain 12915 infection *in vivo* in comparison with that of the tetravalent galabiose compound 'Gal'.

## 2. Experimental Section

### 2.1. Bacterial Strain, Growth Conditions and Inoculum Preparation

*S. suis* serotype 2 strain 12915 was obtained from the strain collection at the Streptococcus Unit at Statens Serum Institut (SSI), Copenhagen, Denmark. It originated from a human case of intestinal bacteremia of *S. suis* serotype 2. The bacteria were stored at −80 °C in ox broth with 10% glycerol (SSI-Diagnostika, Copenhagen, Denmark, cat. no. 1056) until use.

The susceptibility of the *S. suis* serotype 2 strain 12915 to penicillin was investigated by determining the MIC value using the Etest®. The test was performed according to the manufacturer’s instructions (AB BIODISK™, art no. 5100 0268). The susceptibility of the strain was classified according to international break point values for penicillin susceptibility of streptococci (www.abbiodisk.com).

The *S. suis* serotype 2 strain 12915 was cultured from a frozen stock on a 5% horse blood agar plate (SSI-Diagnostika, Copenhagen, Denmark, cat. no. 677) and incubated aerobically overnight (ON) at 37 °C. Todd-Hewitt (TH) (Bacto Difco, cat. no. 249210) broth medium was inoculated with bacteria from the cultured plate and incubated aerobically ON at 37 °C. The tetravalent galabiose compound (‘Gal’, Figure 1) was prepared according to the reported procedure [20]. All media and solutions were prepared according to the manufacturer's instructions. Tetravalent-Galabiose: Ten milliliters of a 0.05% solution of the chemically synthesized tetravalent galabiose compound (Gal) was prepared fresh by dissolving it in 0.9% NaCl (0.5 mg/mL or 186 μM Gal). A solution of Penicillin G (Benzylpenicillin, LEO Pharma) was prepared by dissolving an ampoule of 600 mg (1 million international units, IU) in 6 mL of solvens *ad pen* (SSI-Diagnostika, Copenhagen, Denmark, cat. no. 31486). This was further diluted 10-fold in 0.9% NaCl to obtain a concentration of 10 mg/mL.

### 2.2. Infection Model

Female CFW1 outbreed mice (Harland) 6–8 weeks old were used. These cages were housed in a room with 12 h light/12 h dark periods at a constant room temperature of 21 °C and humidity of 60%. Six mice were kept per cage, and in each experiment the mice were monitored daily for morbidity and mortality. 

Overnight bacterial cultures were suspended and resuspended in 0.9% NaCl and CFU/mL determined. Each mouse was inoculated with 0.30–0.35 mL of inoculum by intraperitoneal (IP) injection. After inoculation the mice were examined once a day over a period of 3 days for signs of clinical disease in order to monitor the infection. On day 3 after inoculation, the mice were sacrificed by cervical dislocation, and the following inner organs were removed: brain, liver, lungs and spleen. After removal, each organ was immediately transferred into separate Falcon tubes with 0.7–0.9 mL physiological saline (0.9 mL for brain and spleen and 0.7 mL for liver and lungs) in order to obtain 1 mL totally. The tubes were refrigerated as soon as possible and preferably put on ice immediately after removal and finally stored at −80 °C. The organs were homogenized using a sterile tissue grinder. Ten-fold dilutions were made in 0.9% NaCl solution and spotted in 20 µL droplets on pre-dried 5% horse blood and TH agar plates. One hundred microliters of the non-diluted homogenate was spread on a 5% horse blood agar plate as a control. All plates were incubated aerobically upside down at 37 °C ON. After incubation, bacterial colonies were counted and CFU/mL calculated.

### 2.3. Characterization of Bacterial Isolates from Animal Organs

Isolated strains from the cultivated mouse organs were all initially identified as *S. suis* by culture on 5% blood and TH agar plates. In order to specifically verify the identity of the chosen isolates as indeed being *S. suis* serotype 2, selected colonies were further characterized by bio- and serotyping methods (data not shown). Serotyping was performed by capillary precipitation test of Lancefield extracts of isolates from IP0, IP1, IP3 and IP4 as described by Slotved *et al.* [30]. Isolates were further biochemically characterized by the automated, commercial kit Rapid ID 32 STREP (bioMérieux, Lyon, France) according to the manufacturer’s instructions. Both methods were also applied to the reference strain of *S. suis* serotype 2 (strain 12915).

### 2.4. Statistics

The logarithm (log10) to the final CFU/mL value for each organ (brain, liver, lung and spleen) from each mouse was plotted in a scatter plot using the Software GraphPad Prism® v. 5.02. 

All scatter plots presenting the results of the mouse experiments were presented with the standard error of the mean values (SEM) as calculated by the GraphPad Prism® software according to the standard statistics. A two-tailed, non-parametric statistical test was performed for each scatter plot to determine if there was a statistical significant difference (P < 0.05) between the groups of mice. If two groups were compared, the Mann-Whitney test was applied to the data, and if more than two groups were compared, the Kruscal-Wallis test was used.

## 3. Results

### 3.1. Infection Model

**Figure 2:**
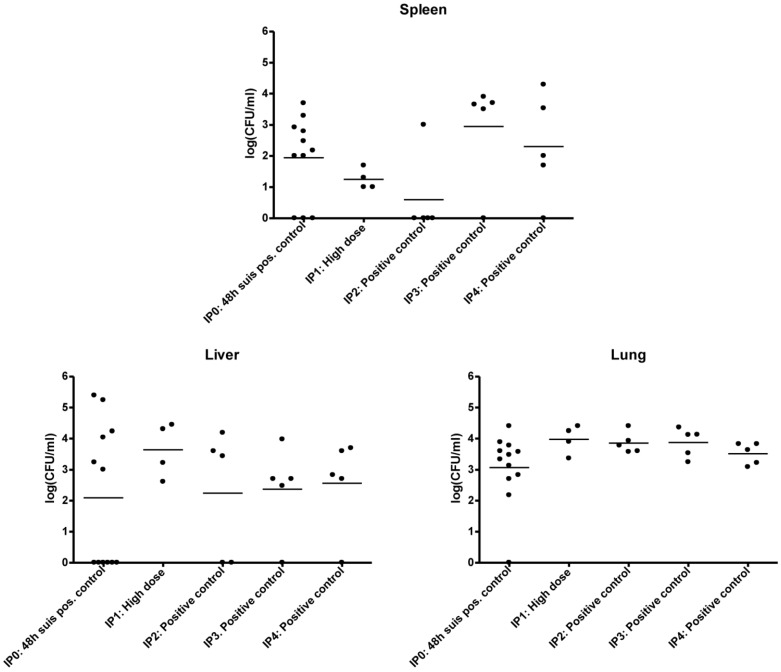
Comparison of positive controls, *i.e.*, without treatment and inoculated with 10^7^ CFU/mL *S. suis* 12915 from mouse experiment IP0-IP4.

*S. suis* infection was established by intraperitoneal injection in mice. Mouse experiments were performed and their time course (IP0, IP1, IP2, IP3 and IP4) are described in detail in Table 1. Infection of the positive control mice, *i.e.*, mice inoculated with *S. suis* only and no treatment, are seen in Table 1, presenting the log CFU/mL values from three different organs of the five conducted mouse experiments IP0-IP4. The mean log CFU/mL values especially from the lungs and livers of all the mouse experiments were similar, whereas the picture was more varied with the spleens. There were no significant difference (P > 0.05) between the means of the five experiments for each plotted organ, and this supported the consistency and quality of the applied peritonitis mouse model (Figure 2). This validates its use for the investigation of the effects of galabiose and antibiotics on the infection. In all experiments (IP0, IP1, IP2, IP3 and IP4) a very low or no infection was detected in the brains (data not presented). 

### 3.2. Experimental Setup (IPO, IP1, IP2, IP3 and IP4)

#### 3.2.1. IP0

IP0 mouse experiments compared the effect of post-treatment with Gal. Group A (control group for Group B) was infected for 24 hours before sacrificing all mice. Group B was infected for 24 hours, and Gal administered 4 hours before sacrificing all mice. Group C (control group for Group D) was infected for 48 hours before sacrificing all mice. Group D mice were infected for 48 hours, with Gal administered 24 hours before sacrificing (Table 1). 

No mice displayed clinical signs of disease on either Day 1 or Day 2 after IP infection with a bacterial dose of approximately 3 × 10^6^ CFU. 

**Table 1:** Overview of Mouse experiments IP0-IP4.

	IP0				IP1			IP2			IP3			IP4		
**Group**	A	B	C	D	1	2	3	A	B	C	A	B	C	A	B	C
	Control to B	24 h	Control to D	48 h	Control	High	Low	Positive control	Pre-treatment	Post-treatment	Positive control	Pre-treatment	Post-treatment	Positive control	Post-treatment	Penicillin treatment
**Number of mice**	12	12	12	12	2	4	4	5	5	5	5	5	5	5	5	5
**Dose volume, mL (inoculum)**	0.30	0.30	0.30	0.30	-	0.30	0.30	0.30	0.30	0.30	0.35	0.35	0.35	0.30	0.30	0.30
**Inoculum conc., CFU/mL**	~10^7 a^				-	6.81 × 10^7^	1.79 × 10^3^	2.23 × 10^7^			1.39 × 10^7^			1.65 × 10^8^		
**Dose volume, mL (Penicillin dose conc. = 10mg/mL)**	-	-	-	-	-	-	-	-	-	-	-	-	-	-	-	2 × 0.30
**Duration of infection**	0–24 h	0–24 h	0–48 h	0–48 h	-	0–72 h	0–72 h	0–72 h	0–72 h	0–72 h	0–72 h	0–72 h	0–72 h	0–72 h	0–72 h	0–72 h
**Gal treatment**	0	20 h	0	24 h	-	-	-	-	÷24 h	24 h	-	÷24 h	24 h	-	24 h	-
**Penicillin** **treatment**	-	-	-	-	-	-	-	-	-	-	-	-	-	-	-	24 h and 48 h

^a^ The exact inoculum concentration was not recorded, but it corresponded approximately to concentrations obtained earlier with the strain incubated under the same growth conditions (*i.e.*, preliminary experiment 2). It was estimated to be in the range of 10^7^ CFU/mL on the basis of this and the observed health status of the infected mice; ^b^ In IP0, IP1, IP2 and IP4 the dose volume of Gal was 0.3 mL of a Gal solution (0.5 mg/mL, 186 μM), thus representing 0.15 mg of Gal. In IP3 was the dose volume 0.35 mL of a Gal solution of the same concentration, *i.e.*, 0.175 mg.

**Figure 3:**
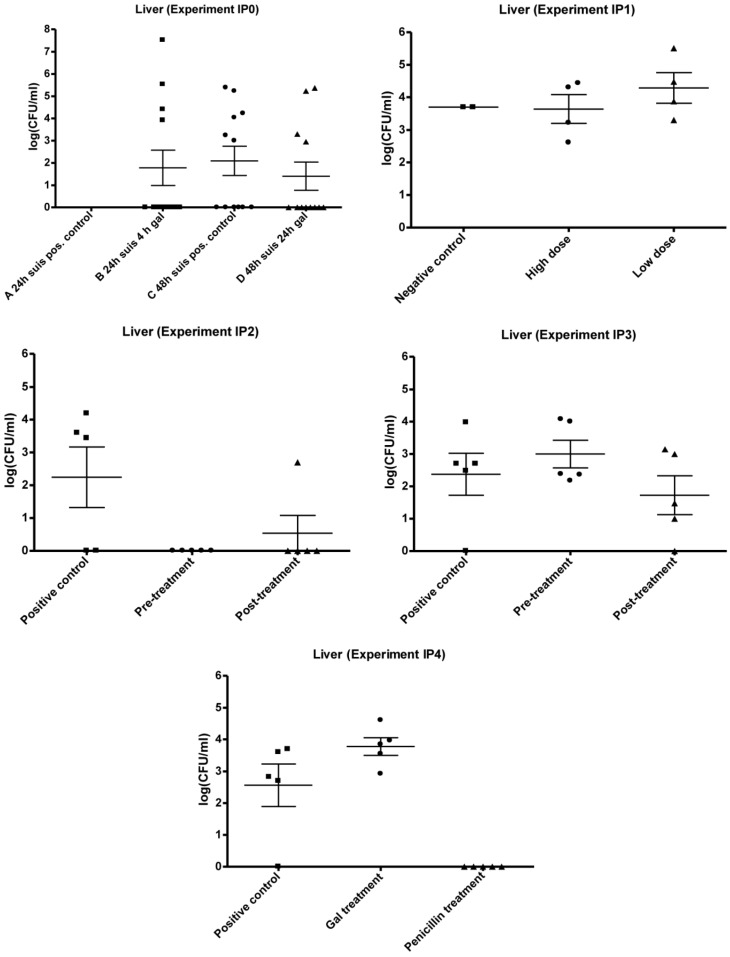
Results of mouse experiment IP0, IP1, IP2, IP3 and IP4: Scatter plots of bacteria detected in liver after cultivation on 5% horse blood agar plates. The values are plotted as log CFU/mL. Group A IP0 not done.

**Figure 4:**
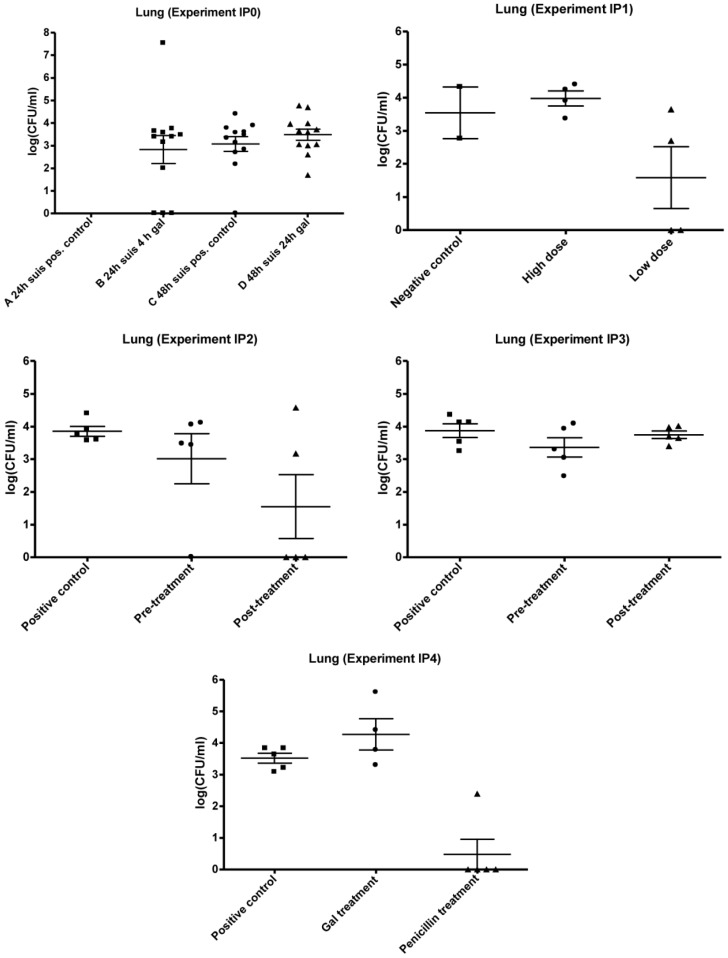
Results of mouse experiment IP0, IP1, IP2, IP3 and IP4: Scatter plots of bacteria detected in lungs after cultivation on 5% horse blood agar plates. The values are plotted as log CFU/mL. Group A IP0 not done.

**Figure 5:**
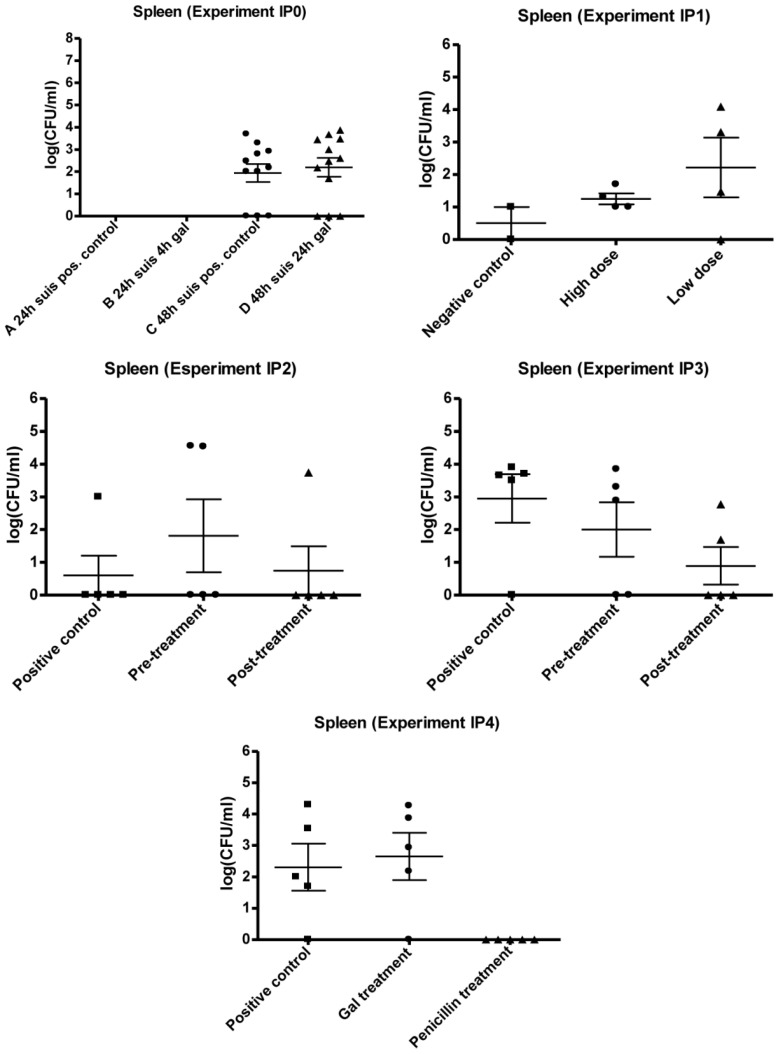
Results of mouse experiment IP0, IP1, IP2, IP3 and IP4: Scatter plots of bacteria detected in the spleen after cultivation on 5% horse blood agar plates. The values are plotted as log CFU/mL. Group A IP0 not done.

The results of organ cultivation from IP0 are presented in Figure 3, Figure 4, Figure 5. Due to experimental problems there is no log CFU/mL data on group A for all organs. Group B, C and D showed similar levels of log CFU/mL when comparing the data within the three organs, and the difference in the mean log CFU/mL for liver, lungs and spleen between Group B, C and D was not significantly different (P > 0.05). The level of infection seems highest in lungs with mean values of 2.9 × 10^6^ CFU/mL (Group B), 4.5 × 10^3^ CFU/mL (Group C) and 1.2 × 10^4^ CFU/mL (Group D). The mean infection levels in the livers were 2.8 × 10^6^ CFU/mL (Group B), 3.7 × 10^4^ CFU/mL (Group C) and 3.4 × 10^4^ CFU/mL (Group D). The lowest mean infection levels were found in the spleens with 8.2 × 10^2^ CFU/mL (Group C) and 1.7 × 10^3^ CFU/mL (Group D). The number of mice (all mice in Group B, C and D combined) with infected livers was lower compared to the number of mice with infected lungs (9–12) and spleens (8–9) ranging from four to six mice.

The results from IP0 did not indicate any inhibiting effect of 'Gal' treatment on the level of CFU/mL within the organs. The general trend in all plots seems to be a constant bacterial level for treated and non-treated groups, as there was no significant difference between means of the control group and the treated groups (P > 0.05).

#### 3.2.2. IP1

Mice were infected with a high and a low dose of *S. suis* strain 12915 in order to define the optimal bacterial dose for the peritonitis mouse model. The dose concentrations and specifications of the setup are shown in Table 1.

At Day 1, 2 and 3 *post* inoculation (p.i.), the mice of the control and low dose groups all appeared healthy, while the mice of the high dose group seemed a little apathetic and affected at Day 1 p.i. On Day 2 their condition seemed to have improved, while complete recovery was observed on Day 3.

The results of the organ cultivation are shown in Figure 3, Figure 4, Figure 5. Overall, bacteria were detected in all isolated organs from mice inoculated with both the high and low dose of strain 12915. Of the organ cultivations, the highest levels of recovered bacteria were in the range of 10^4^ CFU/mL, and predominantly found within the lungs and livers. The mice in the negative control group were found to harbor *S. suis,* and CFU/mL was performed on the detected isolates. However, these counts were suspected to be due to contamination and imprecise plate counting.

Both administration of the low and high dose, of 5.37 × 10^2^ and 2.043 × 10^7^ CFU respectively, resulted in recovery of bacteria within the liver, spleen and lungs, with the highest mean levels detected in the lungs. In the liver and spleen, the mean values of infection seemed to be higher with the low dose compared to the high dose. No statistically significant differences were present between low and high dose groups in any of the organs (P > 0). The results are, however, suspected to be affected by contamination or imprecision.

Since no clinical signs of disease were observed in any of the mice with the high dose, this was chosen for subsequent mouse experiments. 

#### 3.2.3. IP2 and IP3

Mouse experiments IP2 and IP3 were identical except that the dose (volume / inoculum) of Gal was increased from 0.30 mL to 0.35 mL in IP3. The bacterial doses were similar, being 6.69 × 10^6^ CFU in IP2 and 4.87 × 10^6^ CFU in IP3. Both experiments compared three groups of mice: One positive control group (A), one group being subjected to IP Gal treatment before inoculation (B) and one group subjected to IP GAL treatment *post* inoculation (C). In both IP2 and IP3, none of the mice exhibited any clinical signs of illness during the course of the experiments. 

The obtained data of the organ cultivation are presented in Figure 3, Figure 4, Figure 5. *S. suis* was found in three organs. The highest mean level of infection in IP2 and IP3 was found in the lungs, and ranged from 6.1 × 10^3^–9.5 × 10^3^ CFU/mL. In IP2, the second highest mean level of infection was in the liver and in IP3 the second highest mean level was in the spleen. This trend of different infection levels of the organs was similar to the observation in experiments IP0 and IP1. The plots of IP2 and IP3 indicated an inhibiting effect of Gal on the level of *S. suis* in several of the organs (Figure 3, Figure 4, Figure 5). In IP2 the inhibiting effect was observed in the lungs and liver plots, where a decrease in both the number of infected mice as well as in the mean CFU/mL from the positive control to the treated mice seems present (Figure 3, Figure 4). The pre-treated group showed no infection in the liver. In IP3, the possible inhibition effect of Gal on *S. suis* was best presented in the spleen (Figure 5) where a decrease in the mean CFU/mL level from the positive control to the treated groups was observed. 

However, although a decrease in the bacterial level was observed in some of the organs in IP2 and IP3, no significant difference in mean CFU/mL was present between the organs of the control group and treated group (P > 0.05).

#### 3.2.4. IP4

Mouse experiment IP4 compared the effect of antibiotic (Penicillin G) treatment with the effect of Gal treatment on *S. suis* serotype 2 infection. The Gal treatment was performed p.i., and the penicillin treatment was based on data (not shown) from other experiments with the mice peritonitis model performed at SSI involving penicillin treatment of streptococcal infections. The *S. suis* strain 12915 showed a MIC value of 0.125 μg/mL using the Etest®. The bacterial dose administered to the mice in the experiment was 4.95 × 10^8^ CFU. None of the mice in the three groups exhibited any clinical signs of illness throughout the experiment. 

The organ cultivation results of IP4 are presented in Figure 3, Figure 4, Figure 5. The administration of penicillin was effective in treating the infection with *S. suis* serotype 2 strain 12915. Bacteria were present only in the lungs of one penicillin treated mouse. In the rest of the penicillin treated mice, no bacteria at all were detected in any of the removed organs.

With regard to the positive control and the Gal treated group, bacteria were detected in all organs in both groups. The organs harboring the highest mean levels of infection were the lungs with 4.1 × 10^3^ CFU/mL in the positive controls and 1.1 × 10^5^ CFU/mL in the Gal treated group. This was followed by the liver, with mean infection levels of 2.0 × 10^3^ and 1.2 × 10^4^ CFU/mL in control and Gal treated mice, respectively, and the spleen with corresponding mean values of 4.6 × 10^3^ and 5.3 × 10^3^ CFU/mL in control and Gal treated mice respectively. 

The plots showed no inhibiting effect of Gal on the level of bacteria within the organs, as no decrease was observed from the positive control group to the Gal treated group in any of the organs. On the contrary, a higher level of bacteria seemed to be present in the liver, lungs and spleen of the Gal treated group compared to the untreated positive control. There was no significant difference between the positive control group and the Gal treated group in any of the organs (P > 0.05). The penicillin group was significantly lower (P < 0.05) than both the control group and the Gal treated group.

## 4. Discussion and Conclusion

The pathogenesis and virulence mechanisms involved in *S. suis* serotype 2 infections are still unclear, although a number of mouse models have been developed [6,25,26,28,29,31] and despite the fact that *in vitro* assays examining specific virulence mechanisms have been generated [32,33,34,35]. 

Due to rising problems with development of antibiotic resistance, alternative measures to fight and control the infection are increasingly important. The synthetic compound tetravalent galabiose has displayed promising anti-adhesion effects on *S. suis* serotype 2 in *in vitro* assays, but these effects have not yet been examined *in vivo* [20,22]. This study describes a peritonitis mouse model for the investigation of *S. suis* serotype 2 infection *in vivo*, and is the first to examine the effects of synthetic tetravalent galabiose 'gal' treatment of *S. suis* infection *in vivo*.

Since no morbidity and mortality was observed in any of the mice of the experiments involving IP infection with *S. suis* serotype 2 strain 12915 conducted in this study, it was concluded that an IP dose in the range of 10^7^ CFU was suitable for the developed peritonitis model (Table 1). This dose is in agreement with observations in several other studies [28,29]. The dose was also suitable since bacterial cultures were isolated *post mortem* from lungs (primarily), liver, spleen and in a few cases also the brain. This indicates that the dose was high enough to result in dissemination by the bacteria from point of entry throughout the host (resembling meningitis). It was also possible to study the infection over a period of 3 days without disease and mortality. This time span can very likely be prolonged further without development of disease in the animals, and will give the opportunity to study possible effects of repeated infections with *S. suis* serotype 2. Other studies with IP mouse models have involved much longer infection periods with mice surviving several weeks p.i. with *S. suis* serotype 2 strains [28,29,31].

The level of infection upon IP administration of *S. suis* serotype 2 strain 12915 was highest within the lungs followed by a lower bacterial level in the liver and spleen, and the lowest degree of infection was observed in the brain (data not shown). This tendency was consistent and present in all the performed experiments of this study. It indicates a higher affinity of the *S. suis* serotype 2 strain for the lungs compared to the liver, spleen and brain (data not shown) which seems to correlate with the generally accepted opinion of the respiratory tract being an initial site of colonization, and the palatine tonsils acting as the reservoir in healthy carriers [5,36,37] The generally low infection level observed in the brain correlates with the pathogenic mechanism of the bacterium proposed by Gottschalk and Segura [36]. In order to reach the central nervous system, the bacterium must be able to cross the blood-brain barrier (BBB). This may come about by enhancement of the permeability of the BBB by the bacterium, a mechanism that still needs to be described for *S. suis*.

We have developed a peritonitis mouse model for *S. suis* serotype 2 infection with the strain 12915 that can be used for testing treatment regimens and compound interactions against *S. suis* infection (Figure 2). All positive control infections with a dose of ~ 10^7^ CFU/mL showed that there was no significant difference between the infection levels of all five experiments performed. In particular the liver and lungs showed very similar infection patterns. It is generally accepted that the mouse is a good model for *S. suis* infection [1,29], although there have been some studies questioning the mouse model [31]. We believe that this mouse model can be used to examine the effect of a treatment with a potential anti-adhesion reagent. The mouse model in this study also shows a spread of infection throughout the mouse organs, which however seems not to have any severe effects on the behavior of the mouse, thereby giving the benefit of not having to terminate an experiment before scheduled that would interfere with the ethical rules described by animal welfare. 

The Gal treatment of the *S. suis* serotype 2 infection established with the peritonitis mouse model displayed variable results. In preliminary work with the peritonitis and intestinal *S. suis* serotype 2 model in our laboratory (data not shown), Gal appeared to be effective with both oral (intestinal) and the IP model, since inhibiting effects on the bacterial level were observed in liver, lungs and spleen of the IP mice and in the lungs of the orally infected and treated mice. Although not significant, in IP2 and IP3 there was an inhibitory effect of Gal on the bacterial level in the liver (both experiments), lungs (IP2) and spleen (IP3) (Figure 3, Figure 4, Figure 5). In contrast, no inhibitory effect of the compound was observed on the bacterial level of the organs in IP0 and IP4 (Figure 3, Figure 4, Figure 5).

The varying effects of Gal on the *S. suis* serotype 2 infection *in vivo*, observed with the mouse experiments, may be related to capsule expression of the bacterium within the mouse. It has been suggested, that the expression of capsule is regulated according to different stages of infection, in which the encapsulation can be down-regulated during colonization of epithelial cells and up-regulated once in the bloodstream [36]. It has also been proposed that capsule expression influences the accessibility of the adhesins of *S. suis* (P_O_ and P_N_) [38], and adhesion has shown to be increased in the absence of capsular polysaccharide (CPS) [35]. If this thesis holds true, and CPS “hides” the P_O_ and P_N_ adhesins, encapsulated *S. suis* serotype 2 strains will not be inhibited by Gal as the compound will be unable to bind to the bacterium. If the encapsulation is retained after isolation from the mice, the regulation of capsule production can explain the opposing results observed in IP2 and IP3 *versus* IP0 and IP4. The Gal treatment results would then indicate that the isolates of IP2 and IP3 were non-encapsulated *in vivo* in contrast to the isolates of IP0 and IP4, which were encapsulated. 

The lacking effect of Gal may also be due to the applied dosing regimen of the compound during the experiments. Gal was only administered IP once on Day 1 p.i., and the mice were not sacrificed before Day 3. It may be suggested, that the compound had an inhibitory effect on the bacteria right after the administration, but because the organs were not isolated before two days later, bacteria not bound to Gal may have had the chance to adhere to epithelial cells and grow back to the initial level. The effect of Gal would hence not be observed in the isolated organs. In future experiments, this could be investigated by administering several doses of Gal with adequate intervals p.i. in order to ensure a constant level and possible effect of the compound on the infection. A number of other factors regarding the Gal treatment, such as the dose concentration and route of administration, will also have to be tested in future experiments.

This study showed a possible anti-adhesion effect of a synthetic, multivalent galabiose dendrimer on *S. suis in vivo*. In this respect the results can only be compared with the findings of the compound *in vitro*. Effects of Gal on *S. suis*-induced hemagglutination of erythrocytes or bacterial binding to a glycoprotein-functionalized SPR chip were highly promising with low nanomolar inhibition [20,22]. The results of IP4 clearly displayed the effectiveness of penicillin G treatment of *S. suis* serotype 2 strain 12915 infection *in vivo* (Figure 3, Figure 4, Figure 5) in contrast to the Gal treatment, which did not have any effect in this experiment. In the brains, livers and spleens of the penicillin treated mice, no bacteria at all were detected *post mortem*, and in one case only, bacteria were detected in the lungs. On the basis of the Etest® result, these findings were expected and confirmed that our strain is susceptible to penicillin G, and *in vivo* treatment of an infection with the applied dosing regimen is effective. 

Taking these findings together, the study succeeded in applying a peritonitis murine model for the study of infections with *S. suis* serotype 2. The study also showed that the effect with Gal treatment was inconclusive, since experiments IP0 and IP4 displayed no inhibiting effect of Gal, while the two experiments IP2 and IP3 showed a promising effect of the inhibiting of the compound on the level of *S. suis* serotype 2 infection in mice. Due to these opposing results, no final conclusion can yet be made with regard to the anti-adhesion and potential treatment of Gal on *S. suis* serotype 2 infections *in vivo*.

## Acknowledgments

This study was carried out with the financial support from the Commission of the European Communities, specific RTD program Quality of Life and Management of Living Resources, QLK2-CT-2002-01852, POLYCARB. 

## Reference and Notes
